# Heart graft preservation technics and limits: an update and perspectives

**DOI:** 10.3389/fcvm.2023.1248606

**Published:** 2023-11-06

**Authors:** Aurore Ughetto, François Roubille, Adrien Molina, Pascal Battistella, Philippe Gaudard, Roland Demaria, Julien Guihaire, Alain Lacampagne, Clément Delmas

**Affiliations:** ^1^ Phymedexp INSERM, CNRS, University of Montpellier, CHRU Montpellier, Montpellier, France; ^2^ Department of Anesthesiology and Critical Care Medicine, Arnaud de Villeneuve Hospital, CHU Montpellier, University of Montpellier, Montpellier, France; ^3^ Cardiology Department, CHU de Montpellier, University of Montpellier, Montpellier, France; ^4^ Cardio-thoracic and Vascular Surgery Department, CHU de Montpellier, University of Montpellier, Montpellier, France; ^5^ Cardiac and Vascular Surgery, Marie Lanelongue Hospital, Paris Saclay University, Le Plessis Robinson, France; ^6^ Intensive Cardiac Care Unit, Cardiology Department, Rangueil University Hospital, Toulouse, France; ^7^ REICATRA, Institut Saint Jacques, CHU de Toulouse, Toulouse, France

**Keywords:** PGF primary graft failure, MP machine perfusion, ESNP ex situ normothermic perfusion, heart tranplantation, heart preservation, hypothermic machine perfusion (HMP)

## Abstract

Heart transplantation, the gold standard treatment for end-stage heart failure, is limited by heart graft shortage, justifying expansion of the donor pool. Currently, static cold storage (SCS) of hearts from donations after brainstem death remains the standard practice, but it is usually limited to 240 min. Prolonged cold ischemia and ischemia-reperfusion injury (IRI) have been recognized as major causes of post-transplant graft failure. Continuous ex situ perfusion is a new approach for donor organ management to expand the donor pool and/or increase the utilization rate. Continuous ex situ machine perfusion (MP) can satisfy the metabolic needs of the myocardium, minimizing irreversible ischemic cell damage and cell death. Several hypothermic or normothermic MP methods have been developed and studied, particularly in the preclinical setting, but whether MP is superior to SCS remains controversial. Other approaches seem to be interesting for extending the pool of heart graft donors, such as blocking the paths of apoptosis and necrosis, extracellular vesicle therapy, or donor heart-specific gene therapy. In this systematic review, we summarize the mechanisms involved in IRI during heart transplantation and existing targeting therapies. We also critically evaluate all available data on continuous ex situ perfusion devices for adult donor hearts, highlighting its therapeutic potential and current limitations and shortcomings.

## Introduction

Heart transplantation (HT) serves as the gold standard therapy for advanced heart failure (1). The global count of HT procedures has consistently risen each year, surpassing 5,000 cases (2). Nevertheless, this figure remains constrained due to the scarcity of available organ donors, a scenario in which demand far outpaces supply. In France, the shortage of donors remains relatively constant, with only 1 donor for every 2–2.25 transplant candidates (3). This scarcity significantly restricts the viability of HT (4).

Consequently, grafts sourced from extended criteria donors are now under consideration. These donors are typically over the age of 55 years, possess mild left ventricular hypertrophy, exhibit non-obstructive coronary artery disease, are recipients of high doses of vasopressors/inotropes, or show indications of left ventricular dysfunction due to brain death interaction (5–7). In addition, the complexity of recipients is on the rise, with a greater prevalence of comorbidities, redo surgeries, and pre-transplant veno-arterial extracorporeal membrane oxygenation (ECMO). Furthermore, there have been recent alterations to graft allocation strategies in France and the United States of America that are associated with more frequent pretransplant ECMO usage and less favorable outcomes when considering waiting list mortality and post-transplant mortality. However, these effects may not be evident in every dataset and country (8–12). For example, among patients who underwent transplantation between 2010 and 2017, the highest risk of 1-year mortality was linked to the need for end-organ support with ECMO (HR 1.59) and mechanical ventilation (HR 2.11) (13).

The evolving scenario of marginal graft acquisition and the growing complexity of recipients have extended the duration of cold ischemia and subsequent ischemia-reperfusion injury (IRI), both of which contribute to a heightened risk of primary graft failure (PGF) (1, 3). PGF is generally defined by the requirement for high-dose inotropes and/or mechanical support immediately following transplantation. As per the International Society for Heart and Lung Transplantation registry, survival rates diminish with prolonged ischemic periods (14).

Current standard practice involves static cold storage (SCS) for hearts from donors following brain death. This method combines cardioplegia and hypothermia, which substantially reduces the energy needs of the donor heart. Nevertheless, an ischemic time (between aortic clamping in the donor until aortic declamping in the recipient) exceeding 240 min is linked to an elevated risk of PGF (OR 3.01) (15, 16).

Allograft injury can manifest as transient myocardial stunning, which lasts for 12–24 h post-HT (17), or as definitive myocardial stunning (18). However, PGF remains the primary cause of early mortality, accounting for up to 40% of deaths within the initial 30 days after transplantation. The incidence varies between centers based on the definition and acceptance criteria for grafts and ranges from 15% to 40% (3, 19–21).

## Ischemia reperfusion injury in heart transplantation

Currently, the standard approach involves utilizing SCS for hearts obtained following brainstem death (BSD). However, this method is linked to significant occurrences of IRI (16, 18). Recent advancements in understanding IRI have brought this syndrome to the forefront of transplantation discussions. The mechanisms underlying IRI operate at both the organ and cellular level through hypoxia and re-oxygenation processes, which play a substantial role in various pathophysiological processes that contribute to the onset of PGF. Integrating this newfound knowledge about IRI is particularly crucial in the context of HT, which still grapples with prolonged periods of ischemia and the immediate repercussions of IRI on cardiac function upon organ reperfusion.

**Figure 1:**
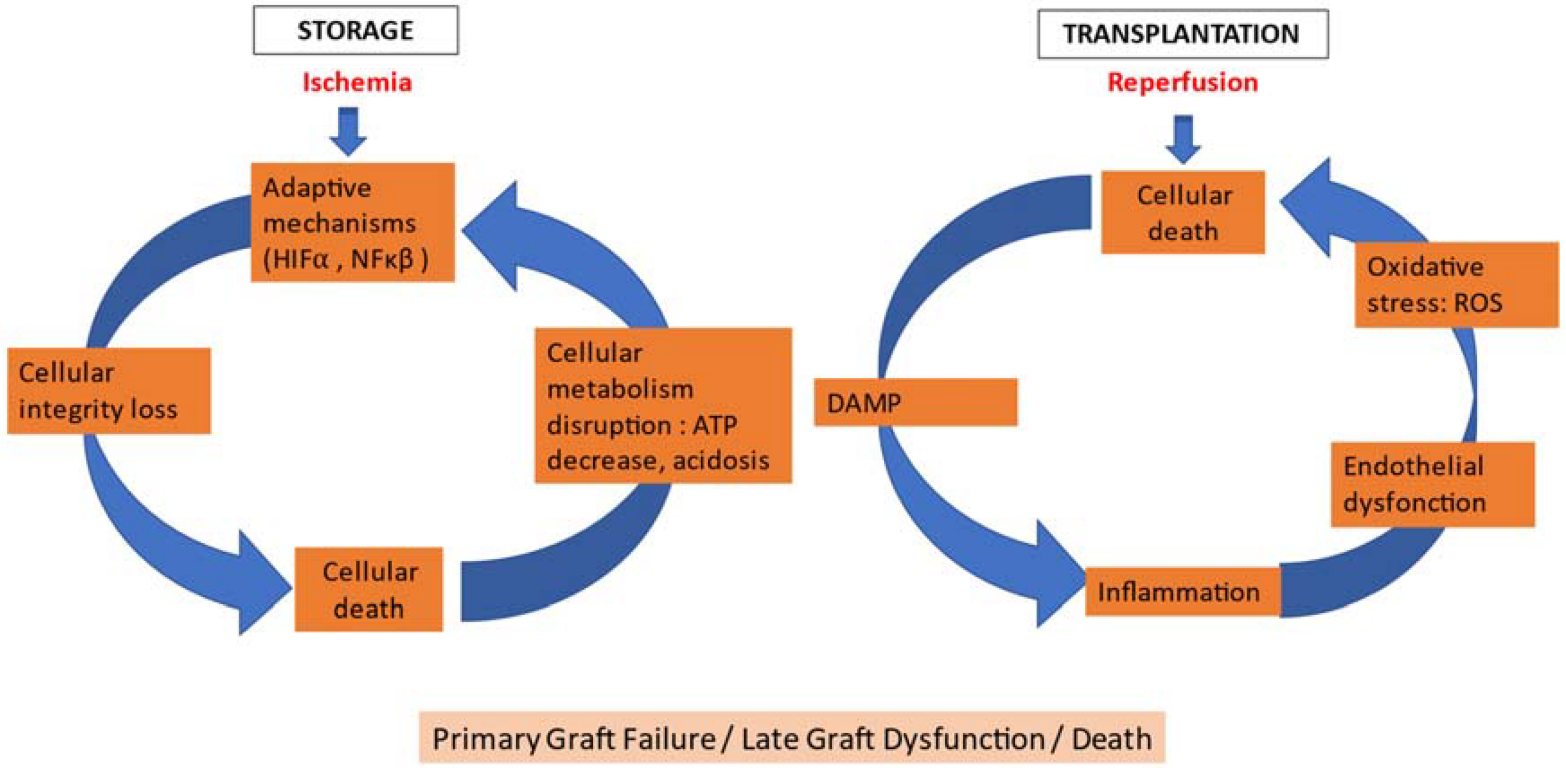
Ischemia-reperfusion process. Ischemia reperfusion: major cellular and clinical consequences in heart transplantation. DAMP: damage-associated molecular pattern; HIF1*α*: hypoxia inducible factor 1*α*; NF*κβ*: nuclear factor k*β*; ROS: reactive oxygen species.

The cellular and molecular mechanisms underlying IRI are intricate and diverse. Among these, impaired mitochondrial function and the depletion of energy metabolites are particularly important for the heart given its close interdependence with energy metabolism. A synthesis of the principal mechanisms and some of their resultant effects is presented in Table 1. It is vital to acknowledge that these processes exhibit high levels of interconnectedness (as illustrated in Figure 1) and are influenced by other factors, such as brain death, donor age, or the pre-existing presence of cardiomyopathy.

**Table 1:** Cellular and molecular processes during IRI.

Damages	Consequences
Oxygen and energy substrate deprivation	High-energy substrates deprivation
Mitochondrial function failure	Damage of oxidative phosphorylation
	Damage of mitochondrial permeability
	Cytochrome C release
	Apoptosis
Ionic damage	Increase of Ca^+^, Na^+^, H^+^
Endothelial damage	Microvascular damage, nitric oxide loss (NO)
Oxidative stress	Reactive oxygen species production
	Protein oxidation
	Immune system stimulation
Inflammation	Immune system stimulation, vasculopathy

Ca^+^, calcium ion; Na^+^, sodium ion; H^+^, proton; NO, nitric oxide.

### Consequences of brain death

Brain death has a significant impact on heart graft function and transplantation. It precipitates an intense systemic inflammatory response often referred to as the “cytokine storm”. This inflammatory response is characterized by an excessive activation of the immune system, leading to the release of inflammatory mediators (cytokines, chemokines, coagulation factors) into the blood.

Myocardial damage resulting from this cytokine storm can compromise the heart's ability to function properly after transplantation. This can manifest as reduced ventricular function, decreased contractility, and an increased susceptibility to IRI during transplantation.

Additionally, the period of brain death may be associated with hemodynamic and metabolic disturbances in the donor, which can also impact cardiac function.

### Ischemia

Ischemia is traditionally defined as a mismatch between the supply of oxygen and energy substrates, and the cellular requirements for proper function and survival. This disparity can lead to various forms of cellular damage. In the context of heart transplantation, the ischemia phase is initiated after the donor's aortic clamp, when the heart is no longer perfused.

#### Mitochondrial damage

In the presence of insufficient oxygen supply, mitochondria are unable to carry out oxidative phosphorylation, a vital process for producing adenosine triphosphate (ATP). Consequently, anaerobic glycolysis becomes the primary means of generating ATP, but the levels are inadequate for fulfilling the cellular energy demands. Prolonged hypoxia results in an ATP deficit that hampers the activity of essential transporters, such as the Na^+^/K^+^-ATPase pump. Consequently, cytoplasmic sodium (Na^+^) levels increase, with a corresponding reduction in potassium (K^+^). This heightened Na^+^ concentration triggers cellular swelling, loss of structural integrity, and the activation of Na+/Ca^2+^ channels, causing an elevation in cytoplasmic calcium (Ca^2+^) levels (22). The excessive influx of calcium due to elevated Na^+^ and inefficient Ca^2+^ removal from the cytoplasm plays a pivotal role in the context of IRI. This surge in calcium activates enzyme systems reliant on calcium, contributing to the initiation of pro-inflammatory processes through the synthesis of lipid mediators, such as prostaglandins. Moreover, this calcium influx triggers the activation of proteases, which disrupt the cytoskeleton and promote cell apoptosis, ultimately leading to cell death.

#### Oxidative stress

Oxidative stress is characterized by an excessive production of reactive oxygen species (ROS), which is often observed during re-establishment of the oxygen supply. The generation of ROS triggers substantial cellular damage, primarily through processes such as lipid peroxidation, which in turn leads to sterile inflammation, causing vascular permeability. Furthermore, ROS production contributes to impairment of mitochondrial function. The modifications in mitochondrial structure, coupled with calcium accumulation and the generation of free radicals, initiate apoptosis via mechanisms involving increased membrane permeability, resulting in opening of the mitochondrial transition pore; the release of cytochrome C; and the subsequent activation of caspase 9, followed by other apoptotic proteases. These processes are modulated by the Bcl-2 protein family situated within the mitochondrial membrane. Nevertheless, when ischemic conditions prevail, adaptive responses to hypoxia manifest (23). An enzyme called xanthine oxidase, which is activated by elevated levels of calcium ions, is responsible for generating superoxide anions under such circumstances. The renowned mechanism relies on the activity of the heterodimeric transcription factor hypoxia-inducible factor 1 (HIF1), which becomes activated in response to decreased tissue oxygen levels. HIF1 triggers the expression of proteins involved in various processes, including erythropoiesis, angiogenesis, and metabolic adaptation.

#### Endothelial damage

The primary target of ischemic conditions is the endothelial cell. The effects of ischemia on endothelial cells significantly impact the vascular damage that contributes to the long-term outcome of heart grafts. These damages arise from various factors, such as hypothermia, elevated intracellular calcium (Ca^2+^) levels, or the composition of graft preservation solutions (e.g., containing high potassium concentrations). Under normal physiological conditions, the endothelium facilitates the release of molecules that regulate the homeostasis of the vascular wall. These molecules include nitric oxide (NO), prostaglandin (PGI2), and endothelium-derived hyperpolarizing factor. However, ischemia disrupts the endothelium's ability to regulate these functions, particularly by altering the expression and activity of nitric oxide synthases (NOSs), leading to a reduction in NO production (24).

Endothelial cells lose their ability to maintain the integrity of the endothelial barrier under ischemic conditions. However, they become activated, resulting in the expression of cytokines, chemokines, receptors, and adhesion molecules that are responsible for recruiting leukocytes. These processes play a crucial role in the formation of lesions during the reperfusion phase (25). Ultimately, prolonged ischemia leads to irreparable cellular damage and disorganization.

#### Inflammation

Brain death and IRI trigger the production of proinflammatory molecules and ROS, which exert a significant impact on graft performance both before and after transplantation (26, 27). The process of brain death leads to the synthesis of proinflammatory molecules (Table 2) during the ischemic phase. These molecules are generated by monocytes, macrophages, and neutrophils that persist in the vasculature following organ removal; endothelial cells and myocardial tissue also contribute to their production (28). Furthermore, even cardiac myocytes can synthesize cytokines during the ischemic period (29). These proinflammatory components continue to be generated during the reperfusion phase, driven by the infiltration of the graft by leukocytes and the activation of neutrophils and macrophages.

**Table 2:** Pro-inflammatory molecules produced by heart transplantation.

Family	Molecules
Cytokines	Interleukins (IL-1β, IL2, IL6, IL-10, IL-12)
	TNF-α
	IFN-y
Chemokines	MCP-1
Adhesion molecules	ICAM-1
	VECAM-1
	PECAM – 1
	Integrin
	L-selectin
Others	iNOS
	Cyclooxygenase -2

IL, interleukin; TNF-α, tumor necrosis factor alpha; IFNy, interferon gamma; MCP-1, monocyte chemoattractant protein-1; ICAM-1, intercellular adhesion molecule-1; VECAM-1, vascular cell adhesion molecule-1; PECAM–1, platelet endothelial cell adhesion molecule-1, iNOS, nitric oxide synthase inducible.

Inflammation can affect the transplanted heart graft via various mechanisms that are complex and multifaceted and could impact overall graft performance and patient outcomes. Here are some possible mechanisms and manifestations of inflammation that can negatively impact graft function: IRI, endothelial dysfunction (leading to reduced NO production, vasoconstriction, increased vascular permeability, and impaired blood flow), leukocyte infiltration (neutrophils and macrophages), myocardial edema (impairing cellular metabolism and ion channel function), allograft rejection (inflammation and immune response leading to tissue damage), fibrosis (impairing cardiac contractility and electrical conduction), cytokine storm (causing widespread tissue damage, hemodynamic instability, and multi-organ dysfunction), microvascular dysfunction (compromising oxygen delivery to cardiac cells), impaired electrical conduction (leading to arrhythmias and conduction disorders), and impaired metabolic balance.

Overall, the impact of inflammation on heart graft function is a complex interplay of immune responses, cytokine signaling, oxidative stress, and cellular interactions. Strategies aimed at mitigating inflammation, such as immunosuppressive therapies and anti-inflammatory agents, may be crucial for maintaining graft function and promoting successful HT outcomes.

### Reperfusion

In the context of HT, the reperfusion phase initiates upon aortic declamping in the recipient, at which point the heart is perfused with warm blood, enabling it to restore contractility.

#### Vascular dysfunction and increased oxidative stress

Reperfusion involves the reinstatement of oxygenated blood supply to the tissue. During this phase, two mechanisms contribute to reperfusion injuries. Firstly, the no-reflow phenomenon arises due to inadequate microvascular reperfusion, often linked to hemostasis disorders marked by increased platelet and complement activation, as well as leukocyte aggregation. Microvascular dysfunction emerges from an imbalance between vasodilator and vasoconstrictor agents. Secondly, the sudden surge in oxygen levels gives rise to an overwhelming production of oxidative stress, surpassing the capacity of cellular antioxidant systems that were already compromised by the preceding ischemic phase. Though oxidative stress is accentuated during reperfusion, its initiation occurs during brain death and is exacerbated by ischemia. The concurrent generation of ROS and NO yields peroxynitrite ions (ONOO-), which are recognized for their cytotoxic effects, as they curtail the availability of NO, a potent vasodilator. The detrimental consequences of reintroduced oxygen also involve the opening of the mitochondrial permeability transition pore, leading to mitochondrial edema, complete inhibition of mitochondrial functions, including ATP production, and the release of cytochrome C. This sequence of events activates the proapoptotic pathway (23). Consequently, IRI stimulates multiple cell death pathways encompassing apoptosis, necrosis, and autophagy.

Though ischemic damage predominantly results in necrosis, reperfusion injury can also trigger apoptosis (25, 30). In a rabbit model, markers of apoptosis detected in reperfused tissue were absent in normal tissue or tissue exclusively injured by ischemia (25). Other investigations have noted the emergence of apoptotic cells within the peri-necrotic region during reperfusion (31, 32).

#### Reperfusion and immune response

The reperfusion phase is also marked by the initiation of an innate immune response within the initial days post-transplantation, followed by an adaptive immune response that plays a role in the development of a chronic immune reaction (33, 34). Thus, in the initial week of reperfusion, the innate immune system orchestrates the recruitment and activation of monocytes, neutrophils, and dendritic cells. This process is facilitated by the generation of immunoreactive molecules during cellular stress, including damage-associated molecular patterns (DAMPs) and ligands for Toll-like receptors (TLRs) expressed within the ischemic tissue; the release of cytokines and chemokines; and the activation of endothelial cells, coupled with the expression of adhesion molecules, such as selectins.

These intricate processes intersect with the recruitment of T cells and the ensuing adaptive immune response, which occurs a few days following reperfusion and intensifies in response to the degree of graft allogenicity (35). This adaptive immune response contributes to the reduction of capillaries and leads to chronic hypoxia, ultimately contributing to delayed graft dysfunction.

### Clinical correlates

Clinical presentations of IRI encompass arrhythmias, microvascular dysfunction, and myocardial stunning. Reperfusion-induced arrhythmias may be influenced by mitochondrial dysfunction. After prolonged ischemia, the mitochondria may struggle to restore or maintain their inner membrane potential, leading to destabilized action potentials and heightened vulnerability to arrhythmias (36).

Myocardial stunning denotes a temporary myocardial dysfunction that arises post-reperfusion. It is thought to stem from a combination of factors, such as oxidative stress, myocardial edema, and persistent calcium overload, even after reperfusion (37). This condition is also intertwined with microvascular injury, and enhanced regional and global myocardial function is observed when the microvasculature remains structurally intact (38–40). As myocardial stunning is reversible over time, short-term administration of inotropic agents can enhance cardiac function and organ perfusion.

Initially, the inflicted damage is reversible and, if blood flow is reinstated during this period, the structures and functions can recuperate to their normal state. However, if ischemia persists for an extended duration, the damage becomes irreparable, culminating in cell death. Before cell death is a vulnerable window during which ischemic myocytes are viable but prone to further injury upon blood flow restoration (i.e., reperfusion injury). The restoration of blood flow triggers three events that can inflict additional harm on myocytes: microvascular obstruction, deposition of red blood cells (hemorrhage), or localized inflammation that could exacerbate microcirculation damage. Myocardial edema, both intracellular and interstitial, exhibits a bimodal pattern following reperfusion. The initial surge occurs shortly after reperfusion and diminishes within a few days, whereas the second wave emerges days later and coincides with the healing process (41).

## Cardioprotective strategies

**Figure 2:**
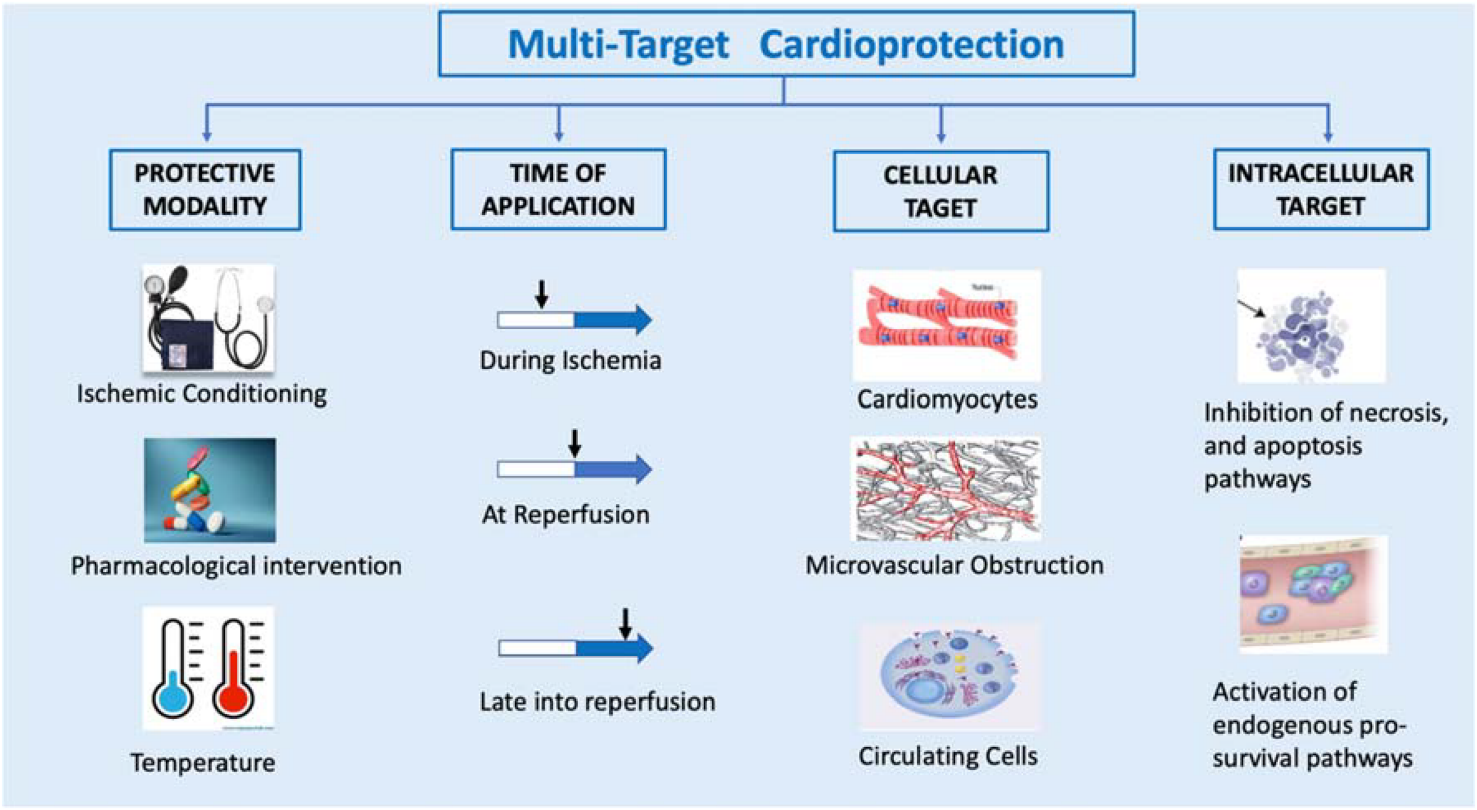
Multi-target cardioprotective strategies.

Over the past few decades, numerous strategies have been proposed to mitigate IRI, capitalizing on a deeper understanding of its mechanisms and its repercussions (42). These strategies can be categorized into different groups based on their protective approach, the timing of their application, their targeted cells, and intracellular components (Figure 2). The principal modes of cardioprotection encompass ischemic conditioning, administration of chemical agents, and the implementation of physical interventions, such as hypothermia. These cardioprotective strategies can also be classified by when they are applied in relation to the ischemic event (i.e., prior to, during, or after) (43). Ideally, treatments that confer protection against IRI should be administered at the earliest opportunity, as the majority of cellular injuries manifest during the initial moments of reperfusion.

### Static graft preservation

#### Hypothermia: static cold storage

SCS is the most widely employed technique for heart preservation. This method involves the prompt removal of blood from the organ, meticulous cleansing of the vascular system with a cold preservation solution and maintaining the heart in a hypothermic state of rest until it is ready for transplantation. Typically, the heart is placed within a sterile bag filled with the preservation solution, then nestled in a container equipped with ice for transportation. These measures are implemented to induce diastolic arrest in the heart, thereby curtailing its metabolic demands and mitigating the adverse impacts of ischemia during transport.

**Figure 3:**
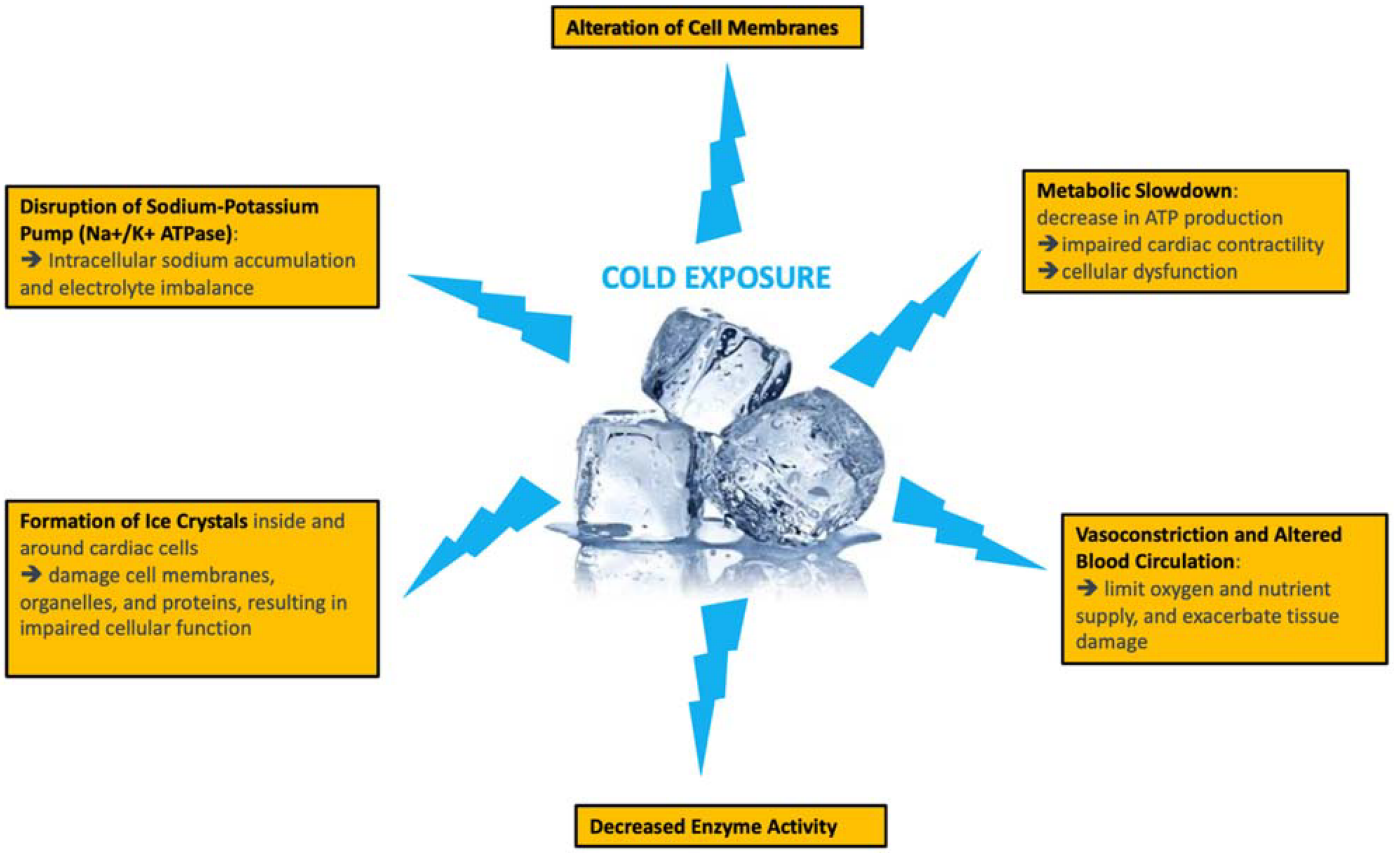
Adverse metabolic consequences of static cold storage.

Although hypothermia does not bring cellular metabolism to a complete halt, it does decelerate the degradation of vital compounds essential for cellular viability (44). Furthermore, hypothermia curtails the rate of lysosomal organelle breakdown within cells, thereby preventing the release of autolytic enzymes and subsequent cell death. As per Vant Hoff’s rule, metabolic activity at 4°C is approximately 10%–12% of the baseline observed under normothermic conditions (45). Thus, the optimal storage temperature is a controversial issue. Low temperatures have obvious advantages in terms of energy reserve preservation but also disadvantages related to cell swelling, increased intracellular concentration of calcium, and cell membrane damage (Figure 3). Hypothermia can lead to impaired Na^+^/K^+^ ATPase activity, resulting in cellular swelling. Under this condition, sodium is unable to be effectively removed and, instead, passively enters the cell. This creates a hyperosmolar intracellular environment, causing water to flow into the cell, resulting in cell edema. To counteract this swelling, colloids are introduced into preservation solutions.

Cold storage within the temperature range of 0–4°C triggers rapid intracellular ATP depletion. Within 4 h, nearly 95% of ATP is hydrolyzed, leading to adenosine monophosphate becoming the predominant nucleotide. This metabolic shift results in acidosis, with the production of two lactic acid molecules (46). The impact of acidosis on ischemic injury is pH-dependent. Profound acidosis activates enzymes, including phospholipases and proteases, causing damage to lysosomes and ultimately resulting in cell death. Thus, effective pH regulation is a vital role of preservative solutions.

SCS has been associated with the promotion of ROS production, likely due to mitochondrial impairment. Free or chelated iron catalyzes the formation of ROS and directly contributes to hypothermia-induced damage by causing mitochondrial dysfunction and initiating apoptosis. ROS rapidly react with other molecules, causing extensive damage to lipids, nucleic acids, and proteins (47). The ensuing mechanism of cell death seems to be dependent on ATP.

The process of rewarming can also induce harmful effects. Systemic collapse and cellular lesions may occur due to the elevation in temperature and oxygen pressure. Upon restoration of blood flow, the oxidation of hypoxanthine and xanthine produces ROS. These free radicals lead to lipid peroxidation, increased membrane permeability, oxidation of membrane proteins, and DNA damage, culminating in enzyme dysfunction. In addition, the accumulated hypoxanthine diffuses out of cells, impeding the cell's capacity to replenish its energy reserves (48, 49). These injuries may be accountable for the delayed recovery of myocardial function, acute graft failure, and long-term coronary atherosclerosis.

Paragonix Technologies (Cambridge, MA) has developed the single-use, disposable Paragonix SherpaPak® Cardiac Transport System for non-perfusing storage. Its purpose is to sustain donor heart temperatures between 4°C and 8°C for extended durations. The system involves suspending the donor heart in a preservation solution, which is de-aired to allow complete submersion of the heart to facilitate even cooling. Subsequently, the inner cannister is inserted into the outer cannister and surrounded by disposable cooling packs. These cooling packs, in contrast to regular ice that undergoes a phase change at 0°C, undergo a phase change at 5°C, thereby maintaining the desired preservation temperatures. In preclinical and clinical studies, various preservation solutions, such as Celsior, University of Wisconsin (UW), and histidine-tryptophan-ketoglutarate (HTK), have been employed with this system (50–52). Preliminary findings from an ongoing large multicenter registry involving 10 sites and 569 patients (comprising 255 ice transports and 314 SherpaPak® transports) have shown favorable early clinical outcomes in the intervention group, including reduced rates of primary graft dysfunction (PGD) and shorter stays in the intensive care unit (53). Thus, this system, which guarantees a stable temperature, provides protection against cold-related injuries, as well as real-time monitoring of data.

#### Ischemic conditioning

Strategies centered around ischemic conditioning encompass both preconditioning and postconditioning. The mechanisms underlying ischemic conditioning remain somewhat elusive due to their multifaceted nature. Ischemic preconditioning (IPC) serves to prevent the uncoupling of NOS and the subsequent generation of reactive oxygen and nitrogen species. In addition, it boosts signaling through proteins, such as protein kinase G, reperfusion injury rescue kinase, and survivor activation factor, in reperfused cardiomyocytes (43). IPC also seems to impact mitochondrial function (54, 55).

Preconditioning's effects can be triggered by pharmacological agents that act on identified targets and have demonstrated protective effects in animal models. Among agents that specifically open mitochondrial K-ATP channels, diazoxide has demonstrated promising outcomes, though it can induce hypotension due to its broad effects. Another potential avenue involves inducing these mechanisms through gene transfer. Gene transfer can be used to induce protective pathways akin to IPC in the context of HT. Certain genes are associated with protective mechanisms that mimic IPC. For example, genes encoding heat shock proteins (HSPs), adenosine receptors, and protein kinase C (PKC) isoforms have been identified as key players in mediating the protective effects of IPC. These genes can be transferred to the graft to induce similar protective pathways. In some cases, gene transfer can also be performed *in vivo*, where the viral vector is directly administered to the recipient after transplantation. This approach allows for targeted gene delivery to the transplanted heart. The transferred genes induce cellular responses that mimic the effects of IPC: reducing oxidative stress, improving cellular energy metabolism, promoting anti-inflammatory processes, and inhibiting apoptotic pathways. By inducing these protective pathways, the transplanted heart becomes more resistant to the detrimental effects of ischemia and reperfusion, leading to improved graft survival, reduced ischemic injury, and enhanced overall function.

However, the practical applicability of preconditioning in clinical settings remains uncertain. Effective administration of the required substances would necessitate their use prior to donor death, which is currently prohibited by ethical and legal considerations. Furthermore, the impact of such treatment would be systemic and could affect all donor organs, potentially compromising the retrieval of specific organs due to their toxicity. A possible alternative is the in-situ administration of pharmacological agents via the preservation solution to replicate the effects of preconditioning.

#### Heart preservation solution

To mitigate the adverse effects of IRI, a range of heart preservation solutions have been developed, each containing varying concentrations of cellular nutrients, metabolites, electrolytes, and antioxidants. The first solution, Euro Collins, was formulated in 1960, followed by the introduction of the UW solution in 1988 (56). Subsequently, modifications and the development of new solutions have grown substantially, with more than 150 different solutions commercialized to date (57, 58). The three most commonly used solutions currently are HTK solution (Perisoc, Khöler Chemie Pharmaceuticals, Germany), UW solution (SPS-1, Poland), and Celsior solution (Institut Georges Lopez, France).

The different solutions for heart graft preservation are designed with specific compositions to optimize the viability and function of the heart during the storage period prior to transplantation. Each solution aims to maintain cellular integrity, prevent IRI, and support the necessary metabolic processes for effective recovery post-transplantation. The main reasons underlying the distinct compositions of heart graft preservation solutions include:

• *Nutrient and electrolyte provision*: Preservation solutions must contain essential nutrients, such as carbohydrates, amino acids, and electrolytes to sustain fundamental metabolic processes and maintain electrolyte balance in cardiac cells during the storage period.
• *Oxygenation and antioxidants*: Preservation solutions may incorporate antioxidant agents to shield cardiac cells from damage caused by free radicals produced during ischemia-reperfusion. Adequate oxygenation in the solution can also support aerobic metabolism of cells.
• *Acid-base balance and pH maintenance*: Preservation solutions need to uphold acid-base equilibrium and appropriate pH levels to prevent metabolic acidosis that can occur during ischemia.
• *Cellular edema reduction*: Some solutions contain osmotic agents to minimize water accumulation in cardiac cells, reducing the risk of cellular edema and structural damage.
• *Inhibition of inflammatory responses and apoptosis*: Certain solutions may include anti-inflammatory agents or substances that inhibit apoptotic pathways to mitigate cellular damage induced by ischemia-reperfusion.
• *Support of mitochondrial function*: Mitochondria are critical for ATP production and cell survival. Some solutions may contain energy substrates and cofactors to support mitochondrial function.
• *Prevention of endothelial injury*: Preservation solutions aim to preserve vascular endothelial integrity to promote adequate blood flow and minimize the risks of thrombosis or endothelial dysfunction.

Ultimately, the compositions of heart graft preservation solutions aim to optimize cellular viability and cardiac function during the storage period to mitigate ischemia-reperfusion-related damage and maximize the success of HT. Each solution may offer specific advantages based on its composition, route of administration, and capacity to protect the heart against various consequences of ischemia and reperfusion.

Numerous studies have attempted to compare post-transplantation outcomes associated with various preservation solutions. However, the results have been inconsistent (59) and have not clearly established the superiority of one solution over another (60–63). In a larger observational and retrospective study, UW solution (*n* = 3,107) demonstrated a significantly higher 1-year survival rate than Celsior solution (*n *= 1,803), though this difference may not have clinical relevance (89.6% vs. 87.0%, *p* < 0.01) (64). Comparative analyses of different preservation solutions are strongly influenced by confounding factors related to extended criteria for organ donation, duration of cold ischemia, complex recipient surgical conditions (e.g., redo surgery and mechanical circulatory support), and the effects of IRI.

Regardless of the preservation solution used, optimal results with SCS are achieved when the ischemia time remains under 4 h. Each additional hour of ischemia beyond 4 h amplifies the risk of graft failure by 43% and increases the 1-year post-transplant mortality risk by 25% (21).

#### Additive drugs during preservation

Targeting cellular signaling pathways that contribute to IRI outcomes is promising as a therapeutic approach. Numerous potential therapies are being explored. Antioxidants and NO transport agents are of particular interest due to the pivotal role of oxidative stress and endothelial damage (48, 49). In addition, drugs that reduce graft immunogenicity before transplantation could help mitigate post-transplantation damage. An alternative approach involves incorporating valproic acid into perfusate, which has had encouraging results (65, 66). The application of metabolomics, proteomics, and genomics techniques to organ preservation offers a promising avenue for identifying new markers of IRI-related damage and subsequent development of novel therapeutic targets.

Anti-inflammatory therapies have also emerged as intriguing targets, though they have been comparatively underexplored in this context. Colchicine, which exerts diverse anti-inflammatory effects by inhibiting neutrophil chemoattraction, the inflammasome network, and pro-inflammatory cytokines, stands out as a potential candidate (67). By disrupting tubulin, colchicine interferes with multiple inflammatory pathways, leading to diminished neutrophil function and reduced migration across the vascular endothelium. Proinflammatory cytokines and adhesion molecule expression are also potential targets for colchicine, thereby impeding local production of coronary chemokines, such as MCP-1 (68), and the secretion of tumor necrosis factor-α by macrophages. Colchicine may even exhibit some anti-fibrotic effects. Various animal studies investigating its cardioprotective effects have found that lower doses of colchicine can inhibit heart apoptosis in rat models (69) and generate anti-fibrotic effects *in vitro* (70), potentially through microtubule cytoskeleton remodeling and/or direct anti-inflammatory actions. Its anti-inflammatory effects are characterized by a reduction in cytokines involved in the post-ischemic inflammatory response, including IL-6, MCP-1, and IL-10 (71, 72).

### Dynamic graft preservation

#### Ex situ machine perfusion

Ex situ machine perfusion was initially proposed to prolong the duration of organ preservation, with the added benefit of potentially expanding the donor pool and improving utilization rates. By supplying the metabolic needs of the myocardium, perfusion can effectively minimize irreversible ischemic cell injury and subsequent cell death. Various heart perfusion systems, categorized as either hypothermic machine perfusion (HMP) or ex situ normothermic perfusion (ESNP), have demonstrated successful preservation of animal and/or human hearts (73). Notably, the longest reported successful human heart preservation time, 16 h, was achieved using ESNP (74).

These perfusion devices are designed to be portable and enable an extended and secure preservation window, facilitating the use of hearts from donors located at a distance. Currently, a single commercially available perfusion system is available for clinical applications, known as the Organ Care System® (OCS, TransMedics, Andovers, USA) (ESNP). In Europe, a multicenter randomized clinical trial is currently underway to explore the safety and potential advantages of the XVivo® technology for heart preservation (XVivo Perfusion AB, Göteborg, Sweden) (HMP) (NCT03991923) (75, 76). The clinical significance of these devices is broad, encompassing an expansion of preservation duration, assessment of extended-criteria donors, and the utilization of re-perfused donor hearts retrieved following controlled circulatory death. Detailed summaries of studies with robust methodologies investigating ex situ machine perfusion are provided in Table 3.

**Table 3:** Complete or recruiting studies investigating *ex vivo* machine perfusion for heart transplantation.

NCT	Type of device	Location	*n*	Protocol	Primary endpoint
NCT03991923	XVIVO	Europe (15 centers)	202	RCT XVIVO heart preservation devices VS cold static storage	30 days mortality and 30 days graft dysfunction
NCT04066127	NIHP	Sweden	66	RCT Non-ischemic heart preservation (NIHP) VS Standard ischemic cold static storage	Survival free of acute cellular rejection and re-transplantation
NCT03831048	OCS	United States	180	RCT OCS heart System VS Cold Storage	Patient survival 6 months post-transplant
NCT03835754	OCS	United States	75	Observational prospective study Single Group Assignment OCS heart System	Patient survival 30 days post transplant
NCT03150147	NIHP	Sweden	47	RCT NIHP VS Ischemic cold static storage	Composite endpoint of patient death due to graft failure, re-transplantation due to graft failure, severe primary graft dysfunction (PGD), need for extra corporal mechanical support such as ECMO within 7 days post transplantation, or acute cellular rejection (ACR) ≥grade 2.
NCT05741723	OCS	United States	276	Observational prospective study Single Group Assignment OCS heart System	Patient survival through 5 years post-transplant
NCT00855712	OCS	United States	128	RCT Organ Care System VS cold static storage	30-day patient survival following transplantation with the originally transplanted heart and no mechanical circulatory assist device at day 30
NCT05047068	OCS	United States	350	Observational prospective study Single Group Assignment OCS heart System	Patient survival at one-year post- heart transplant.

ECMO: extracorporeal membrane oxygenation; OCS: organ care system; RCT: randomized controlled trial.

#### Ex situ normothermic perfusion

##### OCS configuration

Following the retrieval of the heart graft previously preserved using a hypothermic solution, the graft is prepared and positioned on a dedicated module designed for isolated perfusion (see Supplementary Figure S1). The OCS perfusion system consists of a reservoir and an oxygenator, both connected to a centrifugal pump. An aortic cannula is affixed to the graft's aorta and is responsible for perfusing the aortic root and coronary arteries with nutrient-enriched donor blood. An additional cannula is placed in the pulmonary artery to collect blood returning from the coronary sinus, enabling the measurement of blood lactate, which is the only available predictive factor (modest sensitivity and specificity) of myocardial viability to date and, consequently, of the quality of ex situ perfusion (74–77). Lastly, a third cannula is introduced into the left atrium to relieve pressure in the left ventricle.

The anterograde coronary perfusion is regulated by maintaining a minimum pressure of 40 mmHg to ensure sufficient coronary flow and avoid inadequate perfusion at lower pressures (75–77). Pseudo-pulsatility is induced through external pacing of myocardial contractions at a rate of 80 beats per minute. This stimulation aids in emptying the right ventricle, facilitating the collection of coronary venous return while maintaining the capillary bed open and enhancing extracorporeal perfusion (77). A drainage cannula is inserted via the mitral valve to prevent coronary gas embolisms and to offer passive unloading of the left ventricle during preservation.

After the arterial shunt is activated, the heart is gently massaged during the rewarming process. The heart may regain its spontaneous rhythm or fibrillate, necessitating an external defibrillator shock. When electrical activity resumes, the inferior vena cava is tied off. Gradually, the assisted flow is increased to reach a target range of 650–850 ml/min measured at the return to the pulmonary artery, indicating the coronary flow. This is achieved to maintain a desired mean arterial pressure of 70–85 mmHg. Adjustments are made to the mean aortic pressure and coronary flow to ensure the extraction of myocardial lactate, which is evident by a positive arterio-venous difference.

##### Clinical use and applications

Several prospective non-randomized trials comparing SCS with the use of the OCS® demonstrated similar survival rates at 30 days, 1 year, and 2 years (76, 78, 79). The implementation of OCS was associated with extended overall preservation times, reduced cold ischemia duration, and a trend toward decreased PGF rates. In addition to preserving standard donor grafts, clinical interest has been directed towards employing OCS® for marginal donors or cases with long anticipated preservation times. This approach aims to overcome the constraints of the current limited donor pool. The EXPAND trial (80) encompassed grafts with an expected total ischemic time of either ≥4 h or >2 h combined with specific criteria, such as left ventricular hypertrophy, ejection fraction of 40%–50%, circulatory arrest time ≥20 min, or age exceeding 55 years. Eighty-one percent of the included grafts were utilized, resulting in 30-day and 6-month survival rates of 94.7% and 88.0%, respectively. Severe early graft dysfunction within the initial 24 h was observed in 10.7% of cases. Nevertheless, the absence of a comparative group somewhat limits the robustness of these findings and their applicability in clinical practice. Various institutions have independently reported their experiences utilizing OCS® for expanded criteria donors (81). Certain studies have described marginal donor series that were transplanted following OCS preservation, with an average total preservation time of 284 min. In these cases, the 1-year survival rate reached 96.2% (82).

#### Hypothermic machine perfusion (HMP): X VIVO NIHP technology

Initial research efforts in the field of machine heart perfusion concentrated on hypothermic oxygenated perfusion methods (83). Numerous preclinical studies investigating hypothermic oxygenated perfusion have demonstrated the successful preservation of hearts for periods of up to 48 h (84, 85). The XVIVO® Heart Preservation System (XVIVO Inc, Gothenburg, Sweden) (Supplementary Figure 2) is currently undergoing testing in a phase II clinical trial. In contrast to warm graft reperfusion, HMP hinges on the utilization of cardioplegia and hypothermia to induce heart arrest, thereby minimizing metabolic demand during perfusion (75, 86). During this process, the heart is subjected to perfusion with a cold solution (8°C) that is enriched with nutrients and hormones. This cardioplegia solution also includes red blood cells, which serve to restore the depleted ATP levels within myocardial cells, as supported by preclinical studies (87–89).

##### HMP configuration

The HMP procedure involves the use of a serial roller pump, an oxygenator, a leukocyte filter, and a heater/cooler component. To sustain the perfusion circuit, 2.5 L of perfusion solution and 500 ml of compatible irradiated and leuko-reduced donor/recipient blood are required, with a target hematocrit of 18%. The heart is connected to the HMP system using an aortic cannula. Once the heart is in position, the ex-situ circulation process is initiated. Oxygenated blood is introduced into the aortic root, aiming for a perfusion pressure of 20 mmHg and a coronary flow rate of 150–250 milliliters per minute. The heater/cooler mechanism maintains the temperature at 8°C and the pH at 7.4.

##### Clinical use and applications

HMP studies in the context of HT are limited, but they have shown promising results, particularly in the field of lung preservation and evaluation of expanded criteria lung transplant donors. Early clinical applications of the HMP system have also yielded positive outcomes for donor heart preservation. In a nonrandomized open-label phase 2 trial (75), 6-month mortality was lower for patients who underwent HMP compared to those who underwent SCS. The SCS group experienced four deaths within 6 months after transplantation and three cardiac-related adverse events, whereas the HMP group reported no deaths or cardiac-related adverse events. The median preservation times were 223 min for HMP and 194 min for SCS. Importantly, no significant PGF cases were reported. This trial marked the first-in-human study demonstrating the feasibility and safety of normothermic isolated heart perfusion (NIHP) for clinical HT.

Overall, though HMP studies in HT are relatively limited, the outcomes observed thus far are promising and suggest that HMP could play a crucial role in improving donor heart preservation and transplantation outcomes.

#### Ex situ machine perfusion limitations

However advantageous these techniques may be, they are not devoid of substantial limitations. In addition to their high cost and the need for specialized transportation, these methods also demand intricate technical expertise for graft retrieval, device implementation, and oversight during transit as outlined in Table 4. Furthermore, the process of ex situ normothermic perfusion (ESNP) introduces successive bouts of ischemia-reperfusion during organ harvesting and transplantation, potentially heightening the risk of cellular and myocardial damage.

**Table 4:** Comparison of different techniques for heart graft preservation.

	Static cold storage	Transmedics organ care system	XVIVO perfusion XHPS
Price	800–1,000 euros (preservation solution)	30–35,000 euros	Not available
Weight	2.5 Kg	45 Kg	25 Kg
Ergonomics	+++	+	++
Storage temperature	4°C	34–36°C	8°C
Storage duration	4 h	Until 4–6 h	Until 24–48 h
Storage solution	Static	Dynamic	Dynamic
	• 2 L of preservation solution	• 1.5 L of donor leukoreduced blood	• 2.5 L of perfusion solution
		• +500 ml of perfusion solution	• +500 ml of compatible donor/recipient blood irradiated and leukoreduced
Perfusion pressure Coronary flow	–	70–85 mmhg	20 mmhg
	–	650–850 ml/min	150–250 ml/min
Advantages	• Safe organ storage	• Monitor organ function (aortic pressure, coronary flow, heart rate, blood temperature)	• Uses preservation solution
	• Uses preservation solution.		
			• Monitor pO2 and pH of perfusate
	• Cheap		
	• Homogeneous temperature		
			• Allows x-ray
	• Immersed graft	• Housing enables ultrasound assessment and blood sampling	• Console is reusable but Perfusion set is once use
	• Easy to use		
	• No electrical power required		
	• Handling	• Console is reusable but Perfusion set is one time use	• Proven superiority
	• Energy reserves preservation		
Disadvantages	• Ischemic reperfusion injury	• Expensive	• Expensive
	• Short Storage duration	• Needs more staff and qualified personnel	• Needs more staff and qualified personnel
	• Cell swelling	• No functional working mode	
	• Increased Ca2+	• No proven superiority	
	• Damage to cell membranes.	• Successive periods of ischemia-reperfusion	

The current assessment of graft performance during ESNP is confined to observing lactate kinetics and clearance within a perfused, beating heart devoid of afterload conditions. This evaluation approach restricts the comprehensive appraisal of heart function, limiting it to a Langendorf perfusion model, which falls short in terms of sensitivity and specificity. Critical aspects, such as systolic and diastolic functions, cannot be accurately gauged in the absence of preload and afterload considerations, constraining the scope of graft evaluation.

Preclinical studies are imperative to comprehensively understanding the intricate dynamics of IRI across different levels, ranging from the tissue to the microcirculation and endothelium, as well as cellular calcium homeostasis. For example, a porcine model demonstrated a correlation between lactate kinetics and the success or failure of normothermic ex situ preservation, as determined by left ventricular contractility measurements (90). In another study, the normothermic ex situ model exhibited superiority over hypothermic crystalloid solution preservation, demonstrating protective effects by inhibiting apoptosis and oxidative stress in coronary arteries, enhancing both endothelium-independent and endothelium-dependent vasorelaxation, and promoting antioxidant production to prevent ONOO-free radical formation. This approach also resulted in increased ICAM-1 expression in grafts (91). Paradoxically, when comparing three preservation methods after a 30-minute delay in warm ischemia (including warm oxygenated blood, 4°C crystalloid HTK, and a novel HTK-N solute at 4°C), contractility indices were more favorable in the HTK-N and HTK groups compared to the warm blood perfusion group. In addition, immunohistochemical markers, immunoreactivity, and cellular edema were notably reduced in the HTK-N group compared to the warm reperfusion group (92).

Given these complexities, there is a pressing need for additional comparative data to elucidate the inflammatory and immunohistochemical phenomena between ex vivo normothermic perfusion (ESNP) and the conventional cold static preservation approach. Such data could provide a more comprehensive understanding of the underlying processes and aid in refining these preservation techniques.

## Perspectives

### *Ex situ* therapeutic interventions

Ex situ perfusion systems present a significant opportunity to serve as a prominent conduit for the administration of therapeutic interventions to donor hearts without causing adverse effects on other donor or recipient organs. The central objective of these interventions is to mitigate the impact of IRI, with a particular focus on the potential to rejuvenate marginal organs for viable transplantation. Although these therapeutic applications are still in the preclinical phase and have not yet undergone clinical testing, their preliminary outcomes are promising. The prospective integration of ex situ therapies has the potential to not only enhance graft viability, but also contribute to expanding the existing donor organ pool, a prospect that has significant potential for the field of transplantation.

There are several potential therapeutic strategies that can be explored within the ex-situ perfusion context:

- **Pharmacological agents**: Various pharmacological agents, such as antioxidants, anti-inflammatory drugs, and vasodilators, can be administered to the donor heart during ex situ perfusion. These agents aim to counteract oxidative stress, reduce inflammation, and improve vascular function, ultimately protecting the heart from IRI-related damage.
- **Gene therapy**: Gene transfer techniques can be utilized to introduce specific genes into the donor heart during ex situ perfusion. These genes can encode for protective factors or enzymes that counteract IRI processes. For example, introducing genes that promote antioxidant production or inhibit apoptosis could enhance the heart’s resistance to IRI.
- **Cell-based therapies:** Ex situ perfusion systems can facilitate the delivery of therapeutic cells, such as stem cells or genetically modified cells, directly to the donor heart. These cells have the potential to promote tissue repair, regeneration, and immunomodulation, further enhancing the heart’s viability.
- **Metabolic modulation:** Manipulating the metabolic pathways within the donor heart during ex situ perfusion could promote energy production and reduce the negative consequences of metabolic disruption during IRI.
- **Targeted drug delivery:** Nanoparticles and other drug delivery systems can be designed to target specific cellular pathways and deliver therapeutic compounds directly to the heart tissue, minimizing systemic effects and maximizing efficacy.

Though these strategies have shown great promise in preclinical studies, it is important to note that translating them into clinical practice requires rigorous testing and validation. Clinical trials are needed to assess the safety, efficacy, and long-term outcomes of these interventions in human donor hearts. If successful, ex situ perfusion-based interventions could revolutionize the field of organ transplantation by expanding the pool of viable donor organs, reducing the incidence of IRI-related complications, and improving overall graft quality.

#### Blocking apoptosis and necrosis pathways

Interception of the apoptosis pathway in porcine hearts using hypothermic perfusion solutions infused with small interfering RNA molecules targeting key apoptotic and inflammatory enzymes has had notable outcomes in diminishing cellular apoptosis and mitigating myocyte injury. Furthermore, this approach has resulted in enhancement of donor myocardial function (93–95). In a distinct porcine transplantation model, the introduction of oxygen-derived free radical scavengers into the perfusion process has shown the potential to enhance graft functionality while concurrently ameliorating cellular edema (54).

Yet another preclinical investigation unveiled the efficacy of a therapeutic regimen that couples two inhibitors of the necrosis pathway, resulting in a discernible reduction in IRI among rat cardiomyocytes (95, 96). Therapies that foster angiogenesis have also garnered attention. Examples encompass interventions involving the inclusion of vascular endothelial growth factor (VEGF) (97), prokinectin receptor-1 (98), or human multipotent stromal cells (98, 99). These interventions have manifested the capacity to elevate the survival rate of cardiomyocytes subjected to ischemia.

#### Extracellular vesicle therapy

Extracellular vesicles (EVs) derived from various cell lines have exhibited a range of beneficial properties in both *in vitro* and *in vivo* studies, including immunomodulation, antioxidant activity, anti-inflammatory effects, and tissue repair capabilities. These EVs possess the ability to enter target cells and modulate signaling pathways that contribute to tissue healing. EVs have also demonstrated the capacity to hinder left ventricular dilatation and enhance cardiac contractility. At the cellular level, EVs have been effective in mitigating energy depletion, reducing oxidative stress, and curtailing the infiltration of neutrophils and macrophages by activating the protein kinase B and glycogen synthase kinase 3 (AKT/GSK3) pathways. Various animal models have revealed that the administration of EVs prior to, during, or after IRI can counteract inflammatory responses, diminish infarct size, and promote reparative processes (100) while operating independently of circulating cells such as leukocytes and platelets. The use of EVs originating from human lymphoid T cell lines to deliver Sonic hedgehog (SHH) has also demonstrated therapeutic efficacy (101).

In the context of myocardial IRI in murine models, EVs derived from cardiac progenitor cells (CPCs) have demonstrated significant cardioprotective effects (102). When administered intramyocardially following IRI, CPC-derived EVs effectively restrain oxidative stress and myocardial apoptosis by transferring protective mRNA molecules, suggesting that these EVs exert their cardioprotective influence by influencing cellular signaling pathways (103). Collectively, these investigations underscore the potential of diverse EV types in safeguarding the heart from IRI. In the realm of HT, the timing of EV administration offers flexibility, including options such as before ischemia (aortic clamping), during organ preservation (ex situ perfusion), before reperfusion, or post-organ reperfusion. Despite the challenges inherent in this field, several clinical trials are anticipated in the upcoming years to evaluate the therapeutic promise of EVs in patients undergoing solid organ IRI.

#### Donor heart-specific gene therapy

Some studies employing a porcine model of HT with normothermic ex situ perfusion have delved into the utilization of adenoviral vectors and adeno-associated viral vectors for gene administration using the OCS® system (104, 105). The investigations aimed to ascertain the feasibility of employing ex situ perfusion as a conduit for delivering a viral vector to a donor heart during storage, with a focus on evaluating the resultant biodistribution and expression levels of the transgene in the recipient post-transplant. The impact of various components within the OCS®, such as the proprietary solution, donor blood, and ex situ circuitry tubing and oxygenators, was scrutinized. A refined ex situ perfusion strategy optimized for efficient adenoviral vector transduction was employed to introduce 5 × 10^13^ total viral particles of an adenoviral firefly luciferase vector harboring a cytomegalovirus promoter into porcine donor hearts before heterotopic implantation. The comprehensive assessment encompassed levels of expression, protein activity, and dispersion of the firefly luciferase protein, and was conducted over a series of three heart transplants with a post-transplant endpoint of 5 days. Notably, though the perfusion solution and ex situ circuitry had no influence on viral vector transduction, the serum or plasma components of the donor blood significantly hindered the transduction process (104).

Another study within this domain explored the administration of recombinant adeno-associated viral vectors (rAAVs) during normothermic ex situ perfusion to facilitate transgene delivery to porcine cardiac allografts. A myocardially enhanced variant known as SASTG was utilized to assess the transduction efficiency in OCS perfusate. Employing normothermic ex situ perfusion, the introduction of SASTGs containing the firefly luciferase transgene into porcine donor hearts was executed across four heterotopic transplantation procedures. This approach yielded sustained, dose-dependent transgene expression in allografts within 30 days, showing no indications of off-target transgene expression. This investigation underscores the feasibility and efficacy of using AAV vectors during ex situ perfusion to achieve gene delivery to a large animal allograft (105).

Subsequent experiments pertaining to gene delivery to the explanted pig heart involved a method of blood washing prior to the secondary infusion of blood into the perfusate, thereby removing undesired plasma or serum components from the donor blood before its incorporation into the circuit. The enzymatic assessment of luciferase activity within tissues (native heart, allograft, liver, etc.) obtained on days 5 (104) and 30 (103) post-transplant unveiled robust and widespread luciferase activity throughout all sections of the allograft (right and left atria, right and left ventricles, coronary arteries), in contrast to the recipient's native organs. Crucially, luciferase activity in the recipient's heart, liver, lung, spleen, or psoas muscle remained within baseline limits. Luciferase protein expression in the allograft exhibited uniformity and vigor across myocardial areas and coronary arteries.

Taken together, these two investigations collectively establish the feasibility of employing two distinct types of viral vectors to achieve potent and comprehensive transgene expression through administration utilizing the OCS®. This innovative approach to viral vector delivery introduces the prospect of biological modification of the allograft prior to implantation, which could potentially enhance post-transplant outcomes.

### Donation after circulatory death

As the demand for donor hearts continues to rise, transplant centers are increasingly considering the utilization of expanded-criteria donors. This resurgence in interest also extends to the population of donation after circulatory death (DCD), which has the potential to substantially augment the pool of available transplants. Furthermore, DCD can offer grafts from donors who typically possess lower comorbidity levels and are often younger (106). Nonetheless, a primary criticism of this approach revolves around the necessity for the heart to undergo cardiac arrest upon withdrawal of life support, leading to ischemic injury. Several strategies are currently employed and have been investigated, either involving or not involving initial regional normothermic perfusion (NRP), followed by subsequent SCS or normothermic reperfusion using the OCS® during transport prior to transplantation. Nonetheless, a universal consensus regarding the acceptability of NRP has not yet been reached across various countries and transplantation centers.

Encouraging outcomes are also being observed with direct harvesting of DCD hearts combined with ex situ reperfusion.

A recent preclinical study (107) utilizing a porcine model compared three different strategies for heart preservation after circulatory death: (1) normothermic regional perfusion combined with SCS, (2) normothermic regional perfusion combined with oxygenated HMP (X-VIVO® system), or (3) direct procurement combined with oxygenated HMP. Following preservation, HT was performed. The study found that only hearts transplanted in the HMP groups exhibited a significant increase in biventricular contractility 2 h after cardiopulmonary bypass, requiring significantly less dobutamine to maintain cardiac output than in the SCS group. These results provided strong support for the potential applicability of the HMP device in human HT.

Messer and colleagues shared their 5-year experience involving DCD donation, encompassing 79 transplants derived from DCD donors (22 with NRP and 57 with direct harvesting using OCS®). This approach notably amplified the transplant activity of the center by an impressive 48% (164 transplants in total). Survival rates at 30 days [DCD: 97% vs. brainstem death (BSD): 99%; *p* = 1.00] and 1 year (DCD: 91% vs. BSD: 89%, *p *= 0.72) were comparable between the two groups. The US Food and Drug Administration granted approval for application of the OCS® in heart preservation after DCD donation in April 2022, and the results of a dedicated clinical trial (NCT03831048) are anticipated.

In a recent randomized non-inferiority trial, the 6-month survival following transplantation with a resuscitated donor heart assessed via non-ischemic extracorporeal perfusion after circulatory death was on par with survival rates after conventional transplantation involving donor hearts preserved using cold storage after brain death. Notably, 6-month survival was 94% among recipients of a heart from a circulatory-death donor, compared to 90% among recipients of a heart from a brain-death donor (least-squares mean difference, −3 percentage points; 90% CI, −10 to 3; *p* < 0.001 for noninferiority) (81).

Early outcomes stemming from DCD donation have been promising and present a potential avenue for tapping into an underutilized donor pool. Current projections suggest that widespread adoption of DCD donation could elevate the annual count of adult heart transplants in the US alone by 300–600 (108). Given that this avenue has only recently been re-explored, the long-term performance of DCD grafts remains relatively uncharted territory.

## Conclusion

Transplantation stands as the most efficacious approach to addressing end-stage heart disease, yet the insatiable demand for donor organs is anticipated to persistently outpace the available supply. The success of HT hinges upon the preservation of grafts against the detrimental effects of IRI. Capitalizing on the ongoing advancements in techniques and technologies, transplant centers are actively seeking to enlarge the donor pool by pushing the boundaries of established practices and constraints on ischemia time. In this pursuit, the evolution of ex situ perfusion machines has emerged as a pivotal development. These systems offer a dual advantage of enhanced preservation and more precise assessment of organs procured from expanded criteria and donation after circulatory death. Furthermore, the versatility of these ex-situ perfusion platforms extends to their potential as conduits for targeted therapies directed at donor organs prior to transplantation. As the landscape of HT continues to evolve, these innovations promise to redefine the limits of graft viability and optimize outcomes for both recipients and donors.
