# Supplementary material for: Heart graft preservation technics and limits: an update and perspectives
Source: Front Cardiovasc Med. 2023 Nov 6;10:1248606. doi: 10.3389/fcvm.2023.1248606 (PMC10657826; doi:10.3389/fcvm.2023.1248606)
Supplement: Supplementary file 1 [file Datasheet1.pdf]

SUPPLEMENTARY APPENDIX

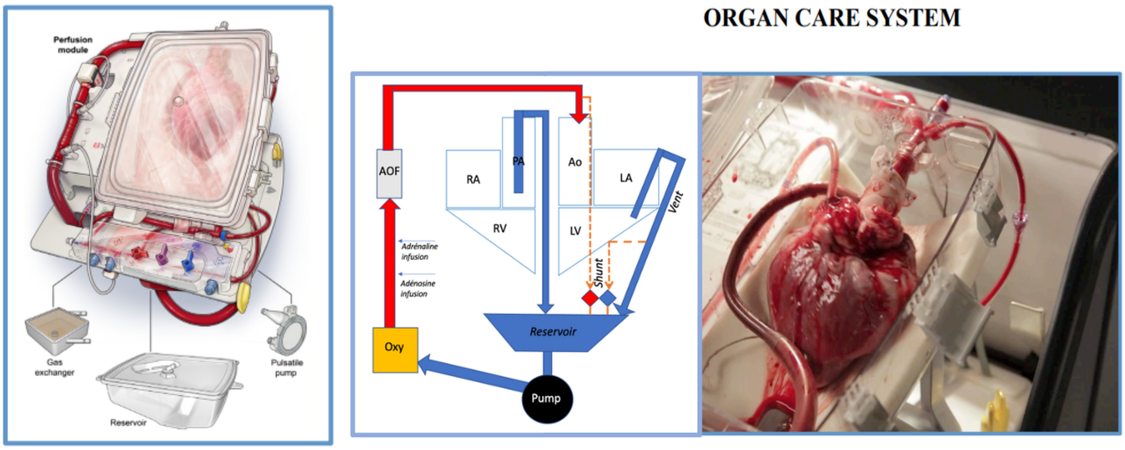

Supplementary Figure 1: Organ Care System (OCS), Transmedics
Ao: aortic root; LV: left ventricle; LA: left atrium; RA: right atrium; RV: right ventricle; AOF: aortic flow.

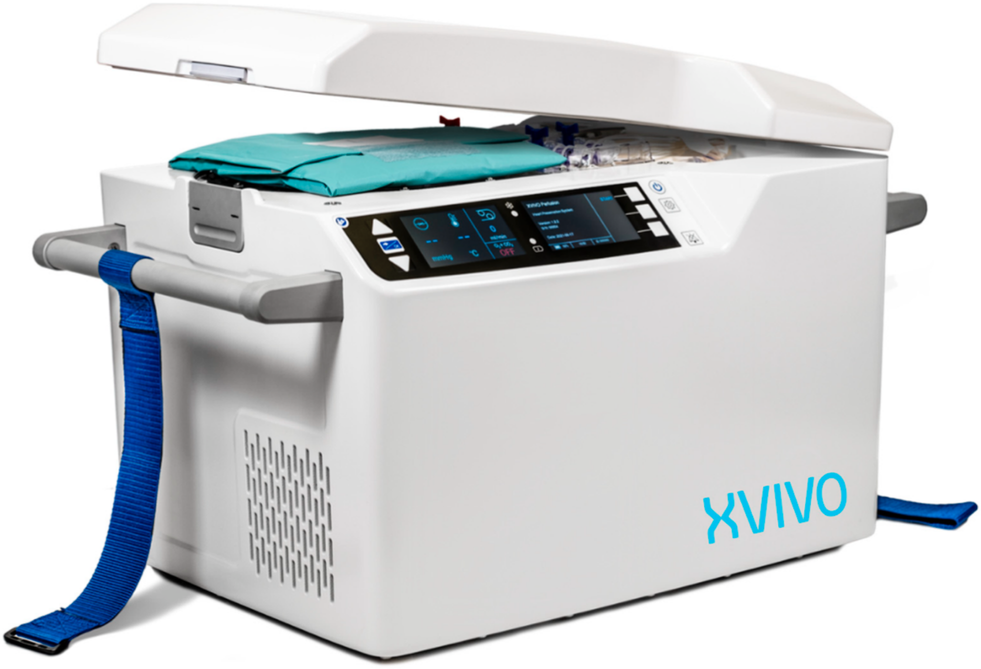

Supplementary Figure 2 : XVIVO Heart Perfusion System.
The heart is submerged in cold blood and cardioplegia solution. Continuous, hypothermic perfusion is established via the aortic root during transport.
